# Effectiveness of Diabetes Community Sharp Disposal Education Module in Primary Care: An Experimental Study in North-East Peninsular Malaysia

**DOI:** 10.3390/ijerph16183356

**Published:** 2019-09-11

**Authors:** Ummu Atiyyah Hasan, Suhaily Mohd Hairon, Najib Majdi Yaacob, Aziah Daud, Anees Abdul Hamid, Norzaihan Hassan, Mohd Faiz Ariffin, Lau Yi Vun

**Affiliations:** 1 Department of Community Medicine, School of Medical Sciences, Universiti Sains Malaysia, Kubang Kerian, Kota Bharu, 16150 Kelantan, Malaysia; 2 Unit of Biostatistics & Research Methodology, School of Medical Sciences, Universiti Sains Malaysia, Kubang Kerian, Kota Bharu, 16150 Kelantan, Malaysia; 3 Primer Unit, Kelantan State Health Department, Kota Bharu, 15200 Kelantan, Malaysia; 4 Kota Bharu District Health Office, Kelantan State Health Department, Kota Bharu, 15200 Kelantan, Malaysia; 5 Non-Communicable Disease Control Unit, Kelantan State Health Department, Kota Bharu, 15200 Kelantan, Malaysia

**Keywords:** diabetes, community sharp disposal, module, effectiveness

## Abstract

*Background*: Structured education is needed to cultivate safe sharp disposal behavior among diabetic patients. Thus, this study aimed to assess the effectiveness of the Diabetes Community Sharp Disposal Education Module in improving knowledge and sharp disposal practice among Malaysian Type 2 diabetic patients. *Methods*: This quasi-experimental study was conducted at primary health clinics in two districts in Kelantan, a state in the North-East Region of Peninsular Malaysia. A total of 132 Type 2 diabetic patients on insulin therapy were involved, with 68 participants in each control and intervention group. The health education intervention was based on the validated Diabetes Community Sharp Disposal Education Module. The knowledge and practices were measured using a validated questionnaire at baseline, one month, and three months after the intervention. *Results*: There was a significant increment in the mean knowledge score for intervention group; from baseline to one month follow up and from baseline to three months follow up [Greenhouse-Geisser; F(1.5, 199.7) = 62.38, *p* < 0.001; effect size (η^2^) = 0.318]. Intervention group had significantly higher mean knowledge score as compared to control group; at one month and three months follow up [F(1, 134) = 17.38, *p* < 0.001; effect size (η^2^) = 0.115]. There was a statistically significant increment in the proportion of participants in the intervention group who practiced the proper community sharp disposal method over time, X^2^(2) = 52.061, *p* < 0.001. *Conclusions*: The Diabetes Community Sharp Disposal Education Module was an effective health education tool to improve knowledge and encourage Malaysian diabetic patients to engage with proper sharp disposal practices.

## 1. Introduction

Type 2 Diabetes Mellitus (DM) is the most common form of diabetes. The World Health Organization (WHO) reported that the global prevalence of diabetes has grown from 4.7% in 1980 to 8.5% in 2014 [1]. Over the past decade, the prevalence has risen faster in low- and middle-income countries, including Malaysia [1,2]. According to a report by the Ministry of Health (MOH), the prevalence of Type 2 DM among Malaysians adults aged 18 years and over had escalated by 50% within a 10 year duration, from only 11.6% in 2006 to 17.5% in 2015 [2,3].

Due to the great therapeutic effect towards achieving optimal glycemic control in Type 2 DM patients as compared to oral hypoglycemic agents, insulin therapy has been recognized as the foundation of diabetes care and is aggressively being prescribed by physicians in Malaysia [4]. The recent National Diabetes Registry, which tracks the care and management of DM in MOH health clinics, reported an increase of over 80% in insulin use within 4 years, from 2009 until 2012 [5]. Patients’ acceptance towards insulin therapy was also driven by the new innovation of an insulin pen with smaller gauge needles to administer insulin, and the availability of convenient home blood monitoring devices [6,7].

All these procedures for diabetes self-care then generate a considerable amount of sharp waste within the household setting. Therefore, Type 2 DM patients, primarily those who are insulin-dependent, are identified as the largest group of patients who use medical sharps on a consistent basis in the community [8,9]. WHO defined sharps as medical devices with sharp points or edges that can puncture or cut skin [8]. In the case of diabetic patients, the sharps commonly used for home treatment are insulin pen needles, insulin syringes, lancets, reusable insulin pens, disposable insulin pens, insulin vials or cartridges, and insulin pump devices [10].

Even though diabetic patients have persistently used medical sharps as part of their daily diabetic care, as reported by previous studies in different countries, their level of knowledge regarding safe community sharp disposal was generally low [7,11,12,13]. The majority of them were not aware of any local safe sharp disposal options in their community, meaning they did not know how to properly dispose of their sharps [7,12]. Knowledge on the possible risks of blood borne disease transmission following unsafe sharp disposal was also low [13]. The postulated reason for this low level of knowledge was never being educated on sharp disposal during regular diabetic consultation [7,11,12,13].

Owing to that, improper sharp disposal practice among diabetic populations is reported to be widespread, especially in most developing countries [12,14,15,16,17]. Large numbers of diabetic patients discard sharps directly into common household bins, burned sharps, or buried them in their household compounds [7,13,15,18,19,20]. Some of them even flushed down the sharps in the toilet, or threw them into old wells, rivers, or canals [19,21]. A relatively small proportion of diabetic patients managed to return their used sharps in proper containers to central collection areas, primarily to health care facilities, for final disposal [11,18,19].

Improperly discarded sharps in the community are of particular concern because of the possible threats and implications they pose to human health and the surrounding environment [16]. A large and growing body of literature shows that unsafe sharp disposal habits increase the risk of the occurrence of needle stick injuries and other sharp related injuries, both to patients themselves and to people near them. Needle stick injuries in the community also increase the risk of blood borne infectious disease transmission later on [22,23]. In addition, improper sharp disposal in household and community settings also accelerates land and water pollution, which can later impose harm to human health as well [16,21,24].

In developed countries like United States, Australia, and United Kingdom, specific ordinances and policies to regulate sharp disposal in community setting are in place to deal with sharp waste generated from home-based care activities [25,26,27]. Standardized community sharp disposal programs and specific education are available to cultivate safe disposal behavior among their chronic disease patients who require self-injecting medication, especially those with diabetes [14,20]. Unfortunately, in Malaysia, sharps waste at the community setting has not been given much attention yet. Until now, no local policy or community sharp disposal program has been made available to handle sharps used by these patients.

As medical sharps waste will be consistently being produced by a growing Malaysian diabetic population, hence, there is a need to develop a structured diabetes education program integrating a locally adapted sharp disposal option, to be offered to these patients. Therefore, this current study aimed to evaluate the effectiveness of the Diabetes Community Sharp Disposal Education Module on improving knowledge and sharp disposal practices among Malaysian Type 2 diabetic patients. The results perhaps can provide evidence on the effectiveness and acceptability of the newly developed module, before it is implemented later on a nationwide scale.

## 2. Materials and Methods

### 2.1. Study Design and Study Setting

This was a quasi-experimental study conducted at government primary health clinics in two selected districts in Kelantan state, which is in the North-East Region of Peninsular Malaysia. The two districts involved were the Pasir Mas and Pasir Puteh districts. Pasir Mas is the second largest district in Kelantan, with 189,292 residents. Meanwhile, Pasir Puteh is the sixth largest district with a total of 116,494 residents [28]. Both selected districts had different numbers of government health centers depending on the sizes of the area covered and the population density in each district. Pasir Mas had 11 primary health clinics and one district hospital, whereas in Pasir Puteh, there were nine primary health clinics and one district hospital. The level of access to health care in both districts was almost equal [29]. Both districts were selected for this study because they were among the districts which recorded the highest number of Type 2 diabetic patients in Kelantan [30]. Only government primary health clinics which had designated diabetic teams that provided primary management for Type 2 diabetic patients were considered in this study. The study was conducted within a nine months duration, from February 2018 until October 2018.

### 2.2. Eligibility Criteria and Recruitment Process

The study involved adults Type 2 diabetic patients who were 18 years old or older [4], who had been using insulin for at least one month, and self-injected their insulin and/or practiced self-monitoring of blood glucose. Patients with gestational diabetes mellitus or established Type 2 diabetes in pregnancy and patients with mental illness were excluded from the study. In each selected Pasir Mas and Pasir Puteh district, simple random sampling using computer generated software was used to select four government primary health clinics which had a diabetic team. At each selected health clinic, purposive sampling was conducted in which only participants who had provided consent were recruited into the study. Participants from Pasir Mas district were assigned to the intervention group while participants from Pasir Puteh district were assigned to the control group, until the required sample size was achieved. The assignment of participants to intervention and control groups based on their district was designed to minimize the possible risk of contamination of information, as the districts were not close to each other.

### 2.3. Diabetes Community Sharp Disposal Education Module

The locally adapted and validated Diabetes Community Sharp Disposal Education Module was developed based on the Health Belief Model by a panel of experts relevant to the scope of the module. They were an epidemiologist, public health medicine specialist in charge of a Non-Communicable Disease and Primary Care Unit from the Kelantan State Health Department, a family medicine specialist, a pharmacist, and a medical statistician. Prior to the current study, a cross sectional study which was conducted among Type 2 diabetic patients in these two selected districts revealed a low to moderate level of sharp disposal knowledge, with almost 99% of patients having improperly disposed of their medical sharp waste [31]. This local evidence-based information served as important point for a need assessment to initiate the newly developed module.

The module was developed following literature reviews and deliberate discussions among the experts to ensure relevant and good content validity. Most of the content in the newly developed module was adapted from local guidelines on diabetes education and insulin injection, as well as guidelines on community sharp disposal from two major established agencies, the United States Environmental Protection Agency (EPA) and Food and Drug Administration (FDA) [25,27,32,33,34,35,36]. All experts agreed that the most suitable community sharp disposal option to be offered in the Malaysian local setting was a supervised drop off container collection site at health clinics. The module therefore consisted of four main topics; (1) medical sharps used for treatment of diabetes in the community; (2) proper handling of sharps prior to disposal; (3) improper community sharp disposal methods; (4) proper community sharp disposal method. The educational materials were in the form of lectures and demonstration, flip chart and pamphlet, which were written in the local Malay language. The module was delivered in two sessions of intervention within a two weeks duration, carried out primarily by the main researcher and assisted by other research team members. All participants in the intervention group received similar interventions based on this module, while those in the control group received a regular diabetes health education.

### 2.4. Malay Version of Diabetes Community Sharp Disposal (M-DCSD) Questionnaire

A validated interviewer-guided questionnaire on community sharp disposal was used to collect data on knowledge and sharp disposal practice from the participants. The original questionnaire was developed by Quiwa and Jimeno [18]. The knowledge domain consisted of three subdomains; proper sharp use, hazards of improper sharp disposal, and a proper sharp disposal method. It was assessed with ten items that had four multiple choice responses and one best answer. A correct answer received a ‘1’ score and an incorrect answer received a ‘0’ score. The total score was converted to a percentage and would range from 0 to 100%. This domain had good psychometric properties. The mean item difficulty index was 0.623, corresponding to a test of moderate difficulty. All the items had a acceptable discrimination index and were point-biserial, with values ranging from 0.22 to 0.43 and 0.31 to 0.53, respectively. The practice domain comprised of two subdomains: the use of a sharp container and methods of sharp disposal. It was assessed with three items with multiple choice responses and more than two correct answers. Details of both domains of the questionnaire can be assessed in the Supplementary Material I. Prior to the current study, the questionnaire was translated and validated into a Malay version.

### 2.5. Follow Up and Assessment

Each participant was assessed based on two outcomes: (1) the total M-DCSD knowledge score, and (2) the proportion of participants who practiced proper community sharp disposal methods at one month and three months after intervention. The interviewer-guided questionnaire was primarily conducted by the main researcher and a research assistant. The research assistant was adequately trained to conduct the interview session, to minimize interrater bias.

The proper community sharp disposal method referred to returning sharp waste at primary health clinics for final disposal. Sharps were collected in either a sharp container approved by the FDA or any heavy-duty plastic household container such as a laundry detergent container, plastic bleach jug or plastic milk container. Such a container has all the basic features of a good sharps disposal container, including being made of a heavy-duty plastic, being able to close with a tight-fitting lid, a puncture-resistant lid that prevents sharps from being able to come out, being upright and stable during use, and being leak-resistant [27,32,35]. For a more objective measurement in the intervention group, an observational checklist form was given to the diabetic team at each participating health clinic. The diabetic team documented in the form each time the participants came to dispose their sharps at the diabetic counter. The appropriateness of the sharp containers and the segregation of sharps from non-sharps waste were also assessed and documented in the form.

### 2.6. Sample Size Determination

The number of participants required was calculated using Stata/SE 14.0 for Windows Software [37] for Repeated Measures Analysis of Variance (RM ANOVA). The mean knowledge score in pre-intervention and standard deviation of the score in pre- and post-intervention were obtained from a previous study [μ1(σ1) = 57.08 (27.84), σ2 = 16.84] [38]. The knowledge score was measured once at baseline, and twice during follow up. A 20% increase in the mean knowledge score was the aim from pre- to post-intervention. With a type I error probability of 5%, type II error probability of 20% (resulting in 80% power of study), and a correlation of 0.5 between baseline and follow-up measurements, the required sample size for this study was 96. Anticipating a 30% dropout rate due to non-responses or missing data, the final required sample size was 136.

### 2.7. Statistical Analysis

The effectiveness of the intervention was estimated with respect to primary outcomes on an Intention to Treat (ITT) basis. Missing endpoint data were imputed using Last Observation Carried Forward (LOCF) method. However, a Per Protocol (PP) analysis approach was also commenced, and the results were compared. Those in the intervention group who did not complete any session of intervention, and those who missed any follow up session or withdrew from the study at any time were considered to be violating the study protocol, and thus were excluded from PP analysis. The effect of intervention on the knowledge score was analyzed using RM ANOVA, assessing within group difference (the time effect), between group difference regardless of time (the overall intervention effect), and between group difference with regard to time (the time-intervention interaction effect). The effect of intervention on community sharp disposal practices was analyzed using Cochran’s Q test and Fisher’s exact test. Statistical analyses were performed using IBM Statistical Program for Social Sciences (SPSS) version 24.0 software (IBM, Armonk, NY, USA).

### 2.8. Ethical Consideration

The study was ethically approved by the Human Research Ethics Committee of Universiti Sains Malaysia (Code: USM/JEPeM/17110663) and the National Medical Research Register (NMMR) Malaysia (Code: NMRR-17-2633-38683(IIR)). All participants gave written consent to participate. The confidentiality of the data was strictly protected.

## 3. Results

### 3.1. Subject Accrual

**Figure 1:**
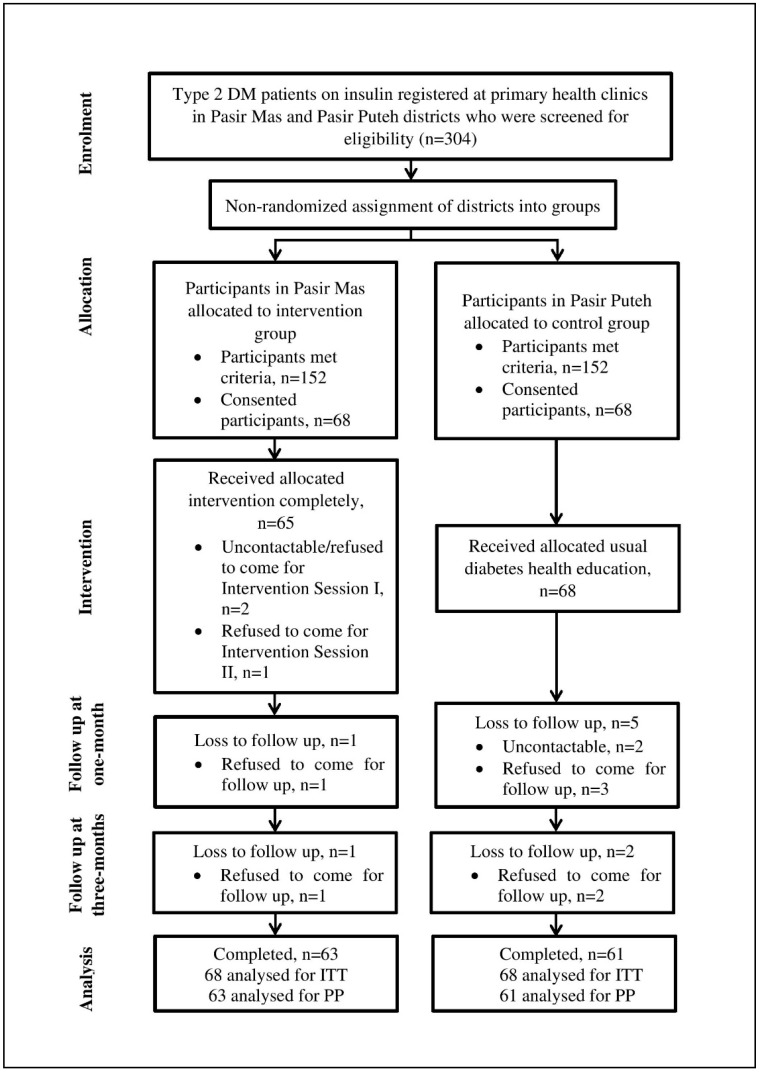
Flow diagram of progress in the intervention study based on Transparent Reporting of Evaluations with Nonrandomized Designs (TREND) statement.

A total of 304 eligible participants were found during two months of the recruitment phase. Of these, 136 participants consented to participate, and thus were recruited into the study. There were 68 participants in the intervention group and in the control group. The follow up for both groups lasted for three months and overall adherence was good with a 91.2% retention rate. 124 participants successfully completed the study. Of these, 63 participants were in the intervention group and the other 61 participants were in the control group. These attrition rates did not differ significantly between the groups. The participant attrition in both groups was due to being uncontactable through telephone (3) or a later refusal to participate or to come for follow up (9). Figure 1 summarized the details of participants’ assignment and random losses to follow up in both groups for the intervention and three months follow up periods.

### 3.2. Groups Characteristics at Baseline

The sociodemographic characteristics of the intervention and control groups did not differ significantly, as shown in Table 1. The patients were predominantly middle-aged, female, married, had a secondary educational background, and were employed.

Both groups also did not differ significantly in their clinical characteristics at baseline. The majority of participants in both groups had diabetes for five years or longer (81.6%), had been taking insulin for less than five years (77.2%), and used a reusable insulin pen (94.1%). Approximately half of participants in each group did self-monitoring of blood glucose at home (55.1%). It was also observed that the characteristics related to community sharp disposal were homogenous in both groups. About one quarter of participants in both groups had received advice on sharp disposal from their health care providers. About one quarter also admitted to causing needle stick injuries before, either to themselves or other family members. The participants in the control group had a slightly higher mean knowledge score of 64.85 (18.81), as compared to the intervention group with 62.06 (19.97), even though the difference did not reach statistical significance. Only 1.5% of participants in each group practiced a proper community sharp disposal method by collecting the sharps in a suitable container and returning them to a health care facility for final disposal. The summary of these findings is presented in Table 2.

### 3.3. Effect of Intervention Using Intention to Treat Analysis

#### 3.3.1. Knowledge Score

For within group difference (time effect), there overall significant changes in M-DCSD knowledge scores were observed over time [Greenhouse-Geisser; F(1.5, 199.7) = 62.38, *p* < 0.001; effect size (η^2^) = 0.318]. Subsequent pairwise comparison with Bonferroni correction showed that there was a significant increase in the mean knowledge score for the intervention group from baseline to one month follow up (Adj. mean difference 22.79; 95% CI: 17.43, 28.16; *p* < 0.001), and from baseline to three months follow up (Adj. mean difference 22.65; 95% CI: 17.19, 28.12; *p* < 0.001). No significant changes in knowledge score were observed in the control group. Table 3 shows the comparison for mean difference of M-DCSD knowledge scores within each group across three-time points.

For between group difference (overall intervention effect), there was an overall significant difference in mean M-DCSD knowledge scores between the intervention and control groups, regardless of time [F(1, 134) = 17.38, *p* < 0.001; effect size (η^2^) = 0.115] (Adjusted mean difference 12.30; 95% CI: 6.47, 18.14; *p* < 0.001). Subsequent pairwise comparison with Bonferroni correction showed that the intervention group had a significantly higher mean knowledge score as compared to the control group at one month follow up (Adj. mean difference 20.29; 95% CI: 13.85, 26.74; *p* < 0.001), and three months follow up (Adj. mean difference 19.41; 95% CI: 13.16, 25.67; *p* < 0.001).

For between group difference with regards to time (time-intervention interaction effect), there was significant interaction between the groups and time in the M-DCSD knowledge score [Greenhouse-Geisser: F(1.5, 199.7) = 61.62, *p* < 0.001; effect size (η^2^) = 0.315]. Table 4 summarized the time effect, overall intervention effect, and time-intervention interaction effect for RM ANOVA of the M-DCSD knowledge score.

#### 3.3.2. Community Sharp Disposal Practice

There was a statistically significant difference in the proportion of participants in intervention group who practiced proper community sharp disposal method over time, X^2^(2) = 52.061, *p* < 0.001. Subsequent pairwise comparison with Bonferroni correction in intervention group showed that there was significant increment in proportion of participants who practiced proper community sharp disposal method; from baseline to one month follow up (*p* = 0.002); from baseline to three months follow up (*p* < 0.001); and from one month follow up to three months of follow up (*p* < 0.001). Meanwhile, for control group, Cochran’s Q test determined that there was no statistically significant difference in the proportion of participants in control group who practiced proper community sharp disposal method over time, X^2^(2) = 0.000, *p* = 1.000. The findings were displayed in Table 5.

At one month and three months follow up, Fisher’s exact test showed the proportion of participants who practiced proper community sharp disposal method in intervention group was significantly higher than in control group, as showed in Table 6.

### 3.4. Effect of Intervention Using Per Protocol Analysis

#### 3.4.1. Knowledge Score

In RM ANOVA using PP analysis, all three effects showed almost similar results to the ITT approach. There were overall significant changes in M-DCSD knowledge scores over time [Huynh-Feldt; F(1.6, 190.1) = 61.56, *p* < 0.001; effect size (η^2^) = 0.335]. There also was an overall significant difference in mean M-DCSD knowledge scores between the intervention and control groups, regardless of time [F(1, 122) = 15.82, *p* < 0.001; effect size (η^2^) = 0.115]. There was significant interaction between the groups and time in the M-DCSD knowledge score [Huynh-Feldt: F(1.6, 190.1) = 60.76, *p* < 0.001; effect size (η^2^) = 0.332].

#### 3.4.2. Community Sharp Disposal Practice

Analysis using the PP approach showed almost similar results as in the ITT approach. Cochran’s Q test showed that there was a statistically significant difference in the proportion of participants in the intervention group who practiced the proper community sharp disposal method over time, X^2^(2) = 52.061, *p* < 0.001. No significant difference was observed in the control group, X^2^(2) = 0.000, *p* > 0.950. Fisher’s exact test also showed that there was a statistically significant difference in the proportion of participants who practiced proper community sharp disposal methods between the control and intervention groups, at one month follow up (*p* = 0.004), and at three months follow up (*p* < 0.001).

## 4. Discussion

The evidence for the use of structured diabetes education programs in diabetes management is growing and has resulted in a positive effect on knowledge and diabetic self-care behaviors [39,40]. However, the evidence of theoretically based diabetes education programs are scanty in Malaysia, and none highlighted medical sharp waste disposal issue in the community. Therefore, the Diabetes Community Sharp Disposal Education Module was developed as a health educational tool to improve knowledge and sharp disposal behavior among Malaysian’s diabetic patients in the community.

### 4.1. Effect of Intervention on Knowledge Scores

The results in this study showed that Diabetes Community Sharp Disposal Education Module was effective in improving the knowledge pertaining to sharp disposal among Malaysian’s diabetic patients. There was a statistically significant increase in sharp disposal knowledge score among participants in the intervention group, from baseline to one month follow up and from baseline to three months follow up.

In this study, the Diabetes Community Sharp Disposal Education Module is a locally adapted education module, using local Malay language with simple and easy layman terms and words. Three different educational instruments were used, as well as a lecture and printed educational tools in the form of a flip chart and pamphlet, with the intention to deliver a systematically structured and repetitive education on community sharp disposal in an interactive way. This locally adapted module with user-friendly instruments helped diabetic patients to absorb the information and thus resulted in a better understanding of it [14,40,41]. On top of that, the intervention session was conducted face-to-face between the researcher and the patients, which further enhanced the two-way communication [14,21,42]. This would explain the increment in the knowledge level of participants in this study. Similar interventional studies conducted among Type 2 diabetic patients in Egypt also revealed an improved literacy level towards sharp disposal in the community, following a structured education delivered by their physicians [38].

The result of this current study showed that knowledge levels among diabetic patients in intervention group substantially increased after the intervention. From one month to three months of follow up, a very slight reduction in the knowledge score was observed, which did not reach statistical significance. This indicated that the information could be sustained for up to three months post intervention. However, the slight reduction warranted the need for continuous and regular education to further sustain the information on sharp disposal, similar to other aspects of diabetes education. This pattern was parallel to recommendations from earlier studies that suggested the community sharp disposal component be integrated into current diabetic education content, and providing education when patients are first being prescribed with insulin [6,14,20,43]. Furthermore, as the results in previous studies showed, the longer patients have diabetes, the more likely that they would experience diabetes burnout [44]. They could become exhausted and frustrated from the long-term use of medications and continuous self-management, thus they might gradually neglect their diabetes self-care [44,45], including the way they handle and dispose of their sharps.

### 4.2. The Effect of Intervention on Community Sharp Disposal Practice

As defined by the FDA and EPA, proper community sharp disposal method refers to the proper use of safe sharp containers and final disposal at correct designated collection centers [27,32,35]. Therefore, the second outcome in this study was determining the proportion of patients using the proper community sharp disposal method, concerning the use of sharp containers which were returned to health care facilities for final disposal. The results showed that Diabetes Community Sharp Disposal Education Module was effective in improving sharp disposal practice among Malaysian diabetic patients. There was a statistically significant increase in the proportion of diabetic patients in the intervention group who practiced proper community sharp disposal method over the three months follow up time.

As underlined in the newly developed module in current study, diabetic patients just need to use their own readily available household containers and discard them at health care facilities, whenever they came to clinic for regular follow up. This option probably incurred no or very minimal cost to them, and was convenient for them to comply with. This was in line with earlier studies that suggested a community sharp disposal program should be simple, affordable, and easily accessible to facilitate compliance [15,20,21,46]. Based on the Health Belief Model, the user-friendly sharp disposal option with a structured community sharp disposal education module in this study acts as a cue or trigger to action, to activate readiness and stimulate overt health-related behaviors for diabetic patients to dispose of sharp waste at proper designated collection centers.

However, despite the provision of education on proper sharp disposal, about half of the patients in the intervention group were observed still not adopting safe sharp disposal practice. Again, using the Health Belief Model as a theoretical framework could help to explain this counterintuitive finding. Cues to action might activate the readiness to change to safer behavior only if the perceived threats and perceived benefits versus perceived barriers are already high [47,48]. Some patients in the intervention group still might not adequately perceive that improperly disposed sharps would later cause environmental pollution and injury to themselves and to others, and need to be adequately convinced that the risk would be substantially decreased by engaging safe sharp disposal behaviors. Because of the relatively low perceived threat, initiatives to change disposal practices might be difficult. Therefore, continuous assessment and reinforcement regarding sharp disposal practice during regular consultation is required [15,21].

As no randomization and blinding were applied in this study, attrition bias would probably occur, especially in the control group, and pose a threat to the internal validity of the study [49]. To minimize this attrition bias, an ITT analysis approach was taken, including by those patients who dropped out in the final analysis. To further assess the impact of non-compliance and those who did not follow up towards the study outcome, an PP analysis approach was also used by including only those patients who strictly adhered to the protocol in the final analysis. However, because of the high retention rate, there were no differences observed in statistical results using both approaches. Therefore, loss of respondents in the current study probably did not produce any effects on the observed outcome.

### 4.3. Study Limitations

Findings of this study highlighted the valuable impact of the locally adapted Community Sharp Disposal Education Module in improving the knowledge and sharp disposal practice among the Malaysian diabetic population. However, the current study did not look into the attitude aspect towards community sharp disposal. Nevertheless, it is imperative to first acknowledge and overcome the barriers preventing safe sharp disposal behavior, because they heavily affected the observed outcome.

### 4.4. Future Research

The replication of this study in various settings or with other populations would be very useful to see the spectrum of community sharp disposal aspect at a larger scale. However, to achieve better understanding about sharp disposal behavior in the community, future research in this area should proceed with a study focusing on a number of other factors, especially attitudes and belief, which also made up the main constructs of the Health Belief Model. A cost-analysis study is also required to evaluate and verify the most cost-effective community sharp disposal option in our local Malaysian setting.

## 5. Conclusions

A locally adapted Community Sharp Disposal Education Module was effective in improving the health literacy and knowledge of sharp use and disposal among the Malaysian diabetic population. The module was also found to be effective in encouraging them to engage with proper sharp disposal practices. Supervised sharp collection at health care facilities as simple and user-friendly community sharp disposal option was proven as feasible for implementation. Since the findings showed convincing results, formal adoption and implementation of the module could be expanded nationwide, to benefit the Malaysian diabetic population at large.

## Figures and Tables

**Table 1:** Comparison of baseline sociodemographic characteristics between intervention and control groups (*n* = 136).

Variables	Frequency (%)			
	Overall (*n* = 136)	Intervention (*n* = 68)	Control (*n* = 68)	*p-*Value ^a^
Age (years)	56.88 (9.35) *	56.43 (10.71) *	57.32 (7.80) *	0.578 ^b^
Sex				
Male	43 (31.6)	24 (35.3)	19 (27.9)	0.356
Female	93 (68.4)	44 (64.7)	49 (72.1)	
Marital status				
Single/Divorced/Widowed	21 (15.4)	10 (14.7)	11 (16.2)	0.812
Married	115 (84.6)	58 (85.3)	57 (83.8)	
Education				
None/Primary level	33 (24.3)	16 (23.5)	17 (25.0)	0.125
Secondary level	85 (62.5)	39 (57.4)	46 (67.6)	
Tertiary level	18 (13.2)	13 (19.1)	5 (7.4)	
Occupation				
Unemployed/Retiree	81 (59.6)	41 (60.3)	40 (58.8)	0.861
Employed	55 (40.4)	27 (39.7)	28 (41.2)	
Monthly household income				
More than RM3000	22 (16.2)	14 (20.6)	8 (11.7)	0.293
RM1000–RM3000	70 (51.4)	35 (51.5)	35 (51.5)	
Less than R1000	44 (32.4)	19 (27.9)	25 (36.8)	

* Mean (SD). ^a^ Chi-square test. ^b^ Independent *t*-test.

**Table 2:** Comparison of baseline clinical and sharp disposal characteristics between intervention and control groups (*n* = 136).

Variables	Frequency (%)			*p-*Value ^a^
	Overall (*n* = 136)	Intervention (*n* = 68)	Control (*n* = 68)	
Duration of diabetes				
5 years or more	111 (81.6)	55 (80.9)	56 (82.4)	0.825
Less than 5 years	25 (18.4)	13 (19.1)	12 (17.6)	
Duration of insulin use				
5 years or more	31 (22.8)	17 (25.0)	14 (20.6)	0.540
Less than 5 years	105 (77.2)	51 (75.0)	54 (79.4)	
Insulin injection form				
Disposable insulin pen only	5 (3.7)	3 (4.4)	2 (2.9)	0.758 ^c^
Reusable insulin pen only	128 (94.1)	63 (92.7)	65 (95.6)	
Both disposable and reusable insulin pen	3 (2.2)	2 (2.9)	1 (1.5)	
Self-monitoring of blood glucose				
No	61 (44.9)	33 (48.5)	28 (41.2)	0.389
Yes	75 (55.1)	35 (51.5)	40 (58.8)	
Previous advice on sharp disposal from health care providers				
No	94 (69.1)	49 (72.1)	45 (66.2)	0.458
Yes	42 (30.9)	19 (27.9)	23 (33.8)	
History of needle stick injury to self or family members				
No	103 (75.7)	50(73.5)	53 (77.9)	0.548
Yes	33 (24.3)	18 (26.5)	15 (22.1)	
M-DCSD knowledge score	63.46 (19.38) *	62.06 (19.97) *	64.85 (18.81) *	0.402 ^b^
Disposing sharps at health care facilities				
No	119 (87.5)	57 (83.8)	62 (91.2)	0.195
Yes	17 (12.5)	11 (16.2)	6 (8.8)	
Proper community sharp disposal method				
No	134 (98.5)	67 (98.5)	67 (98.5)	1.000 ^c^
Yes	2 (1.5)	1 (1.5)	1 (1.5)	

* Mean (SD). ^a^ Chi-square test. ^b^ Independent *t*-test. ^c^ Fisher’s exact test.

**Table 3:** Comparison for mean difference of M-DCSD knowledge score within each group (time effect), using the Intention to Treat (ITT) approach (*n* = 136).

Comparison	Intervention (*n* = 68)		Control (*n* = 68)	
	Adjusted Mean Diff. (95% CI)	*p*-Value	Adjusted Mean Diff. (95% CI)	*p*-Value
Baseline to one month follow up	22.79 (17.43, 28.16)	<0.001	−0.29 (−3.89, 3.31)	>0.950
Baseline to three months follow up	22.65 (17.19, 28.12)	<0.001	0.44 (−3.42, 4.31)	>0.950
One month to three months follow up	−0.15 (−1.11, 0.81)	>0.950	0.74 (−2.88, 4.35)	>0.950

RM ANOVA within group analysis was applied [Greenhouse-Geisser; F(1.5, 199.7) = 62.38, *p* < 0.001; effect size (η^2^) = 0.318] followed by a pairwise comparison with a confidence interval adjustment.

**Table 4:** Time effect, overall intervention effect and time-intervention interaction effect for RM ANOVA of the M-DCSD knowledge score, using an ITT approach (*n* = 136).

Time	Adjusted Mean (95% CI)		*p*-Value	
	Intervention Group	Control Group	Time-Intervention Interaction Effect ^c^	Overall Intervention Effect ^b^
At baseline	62.06 (57.41, 66.71)	64.85 (60.20, 69.51)	0.402	<0.001
At one month follow up	84.85 (80.30, 89.41)	64.56 (60.00, 69.12)	<0.001	
At three months follow up	84.71 (80.28, 89.13)	65.29 (60.87, 69.72)	<0.001	
Time effect *p*-Value ^a^	<0.001	0.884		

^a^ RM ANOVA within group analysis was applied [Greenhouse-Geisser; F(1.5, 199.7) = 62.38, *p* < 0.001; effect size (η^2^) = 0.318] followed by pairwise comparison with confidence interval adjustment. ^b^ RM ANOVA between group analysis was applied [F(1, 134) = 17.38, *p* < 0.001; effect size (η^2^) =0.115] followed by pairwise comparison with confidence interval adjustment. ^c^ RM ANOVA between group analysis with regard to time was applied [Greenhouse-Geisser: F(1.5, 199.7) = 61.62, *p* < 0.001; effect size (η^2^) = 0.315].

**Table 5:** Comparison in proportion of proper community sharp disposal method over time in intervention and control group, using ITT approach (*n* = 136).

Treatment Group	Comparison	Disposal Method		X^2^(2) (df)	^a^*p*-Value
		Improper	Proper		
Intervention	Baseline	67	1	52.061 (2)	<0.001
	One month follow up	57	11		
	Three months follow up	34	34		
Control	Baseline	67	1	0.000 (2)	1.000
	One month follow up	67	1		
	Three months follow up	67	1		

^a^ Cochran’s Q test.

**Table 6:** Comparison in proportion of proper community sharp disposal method between control and intervention group at one month and three months follow up, using ITT approach (*n* = 136).

Comparison		Sharp Disposal Method		^a^*p*-Value
		Improper	Proper	
One month follow up	Control	67	1	0.004
	Intervention	57	11	
Three months follow up	Control	67	1	<0.001
	Intervention	34	34	

^a^ Fisher’s exact test.

## Supplementary Materials

The following are available online at https://www.mdpi.com/1660-4601/16/18/3356/s1, Supplementary Material I: Diabetes Community Sharp Disposal (M-DCSD) Questionnaire.
