# Supplementary material for: Effectiveness of Diabetes Community Sharp Disposal Education Module in Primary Care: An Experimental Study in North-East Peninsular Malaysia
Source: Int J Environ Res Public Health. 2019 Sep 11;16(18):3356. doi: 10.3390/ijerph16183356 (PMC6765895; doi:10.3390/ijerph16183356)
Supplement: Supplementary file 1 [file ijerph-16-03356-s001.pdf]

A. Read the questions carefully. ENCIRCLE the answer that in your opinion is the most appropriate.
Choose only ONE answer per item and please leave no question unanswered.

1. You plan to reuse your insulin syringe. What should you do after using it for the first time?
    - A) Put the cap back on
    - B) Clean the needle with alcohol
    - C) Store the syringe in the freezer
    - D) Sterilize the needle with a flame

2. While injecting insulin, the insulin syringe slips out of your hand and falls to the ground. What should you do?
    - A) Use the syringe that fell but clean it with alcohol first
    - B) Wipe the syringe with a clean cloth then use it
    - C) Throw away the syringe and use a new one
    - D) Just use the syringe like nothing happened

3. Which infectious disease can you get after being accidentally pricked by a syringe used by another person?
    - A) Dengue
    - B) Tetanus
    - C) Cancer
    - D) Hepatitis

4. Who of the following persons in the community is at greatest risk of getting accidentally pricked by used insulin syringes?
    - A) Garbage scavengers
    - B) Midwife
    - C) Teacher
    - D) Street vendor

5. You have young children in your home and you are concerned that they might play with your used or unused insulin syringes. Where is the best place to store your insulin syringes?
   A) In the refrigerator, along with your insulin
   B) In a high cabinet that is hard to reach
   C) In a hidden place like underneath your clothes
   D) In a drawer without a lock

6. If you inject insulin while outside the house, what should you do with the used insulin syringe?
   A) Throw them in the nearest garbage can
   B) Flush them down a toilet
   C) Bring them back home
   D) Throw them in the street

7. Used insulin syringes are best
   A) Recycled like newspaper and bottles
   B) Burned in the backyard
   C) Given to other diabetics who use insulin so they can reuse them
   D) Given to the garbage man for disposal

8. Which of the following is the best way to dispose of used insulin needles and syringes?
   A) Flush them down the toilet
   B) Bury them in the ground in your backyard
   C) Place them in a puncture-resistant container and dispose with household waste
   D) Burn them in your backyard

9. What is the best container for storing used insulin syringes or needles safely?
   A) Plastic bottle
   B) Glass bottle
   C) Plastic bag
   D) Metal can with no cover

10. What should you do before throwing away your used insulin syringe?
    A) Pull out the needle using pliers
    B) Cover the needle with the cap
    C) Bend the needle
    D) Cut the needle with scissors

B. Put a check mark (√) on the box (□) of your answer of choice.

1. Which of the following are the methods you use in disposing your used insulin syringes? (You can choose more than one answer)
   - □ Thrown directly into the garbage can mixed with the household trash
   - □ Thrown into the garbage can but placed in a container like a bag or bottle
   - □ Placed in a container like a bag or bottle and given to the garbage man
   - □ Buried in the ground
   - □ Burned
   - □ Others (specify) ______________________________

2. Where do you place your used insulin syringes just before disposing them? (You can choose more than one answer)
   - □ No container used
   - □ Plastic bag
   - □ Paper bag
   - □ Plastic bottle
   - □ Glass bottle
   - □ Metal container (e.g. biscuit can)
   - □ Wrapped in paper
   - □ Others (specify) ______________________________

3. Do you inject insulin when you're outside the house?
   - □ No
   - □ Yes

   If yes, how do you dispose of your used insulin syringes?
     - □ I bring them home
     - □ I throw them in any available trash can
     - □ I throw them anywhere
     - □ Others (specify) __________________________
